# Effect of oregano essential oil on intestinal immunoglobulin G in Holstein dairy bulls

**DOI:** 10.3389/fvets.2024.1382396

**Published:** 2024-07-29

**Authors:** Meiling Xu, Wangdong Zhang, Fanyun Kong, Baoshan Wang, Jing Pan, Jinping Shi, Qiyan Liu, Pengjia He, Yue Ma, Qiang Cheng, Zhao Zhang, Zhaomin Lei

**Affiliations:** ^1^College of Animal Science and Technology, Gansu Agricultural University, Lanzhou, China; ^2^College of Veterinary Medicine, Gansu Agricultural University, Lanzhou, China; ^3^Jingchuan County Animal Husbandry and Veterinary Center, Pingliang, China; ^4^Jing Chuan Xu Kang Food Co., Ltd., Pingliang, Zhangye, China; ^5^Gansu Huarui Agriculture Co., Ltd., Zhangye, China

**Keywords:** oregano essential oil, Holstein dairy bull, IgG, jejunum, ileum, colon, oregan

## Abstract

**Introduction:**

Immunoglobulin G (IgG) is important in mediating humoral immunity and in the maintenance of immune homeostasis in the intestinal mucosa. Oregano essential oil (OEO) is a natural herbal extract that possesses antimicrobial, antioxidant, anti-inflammatory, and immunomodulatory properties. As the effects of OEO on intestinal mucosal immunity in Holstein dairy bulls remained unclear, we investigated the effect of dietary supplementation of OEO on IgG levels and IgG^+^ cells residing in the intestinal tract in Holstein dairy bulls.

**Methods:**

Twelve Holstein bulls in good health of approximately 10 months of age were selected for the experiment and randomly equally divided into two groups. The control (CK) group was fed a basal ration, and in the OEO group, the basal ration was supplemented with OEO (20 g/head/day). After 300 days of feeding, tissue samples of the jejunum, ileum, and colon of the bulls in each group were collected for histopathological analysis, immunohistochemistry, and enzyme-linked immunosorbent assays, respectively.

**Results:**

The jejunum, ileum, and colon of bulls in the CK group had obvious pathological damage, whereas the structure of each intestinal segment was clear and intact. In the OEO group, pathological damage was significantly reduced. IgG^+^ plasma cells were diffusely distributed in the lamina propria of the jejunum, ileum, and colon in the CK and OEO groups, with no significant difference between the groups. OEO supplementation significantly reduced the number of IgG+ plasma cells in each intestinal segment, with the highest decrease rate being noted for the ileum (22.87%), followed by the colon (19.45%) and jejunum (8.52%). ELISA test results and immunohistochemical results were mutually verified. The change in IgG content was consistent with the trend of change in the number of IgG^+^ plasma cells.

**Discussion:**

Our findings suggest that OEO supplementation does not alter the diffuse spatial distribution of IgG^+^ plasma cells in the intestines of Holstein dairy bulls, but lowers immunoglobulin levels to normal levels, significantly reduces intestinal damage, and may enhance mucosal immune defence barrier function by inhibiting inflammatory reactions.

## 1 Introduction

The intestinal tract not only is a key site for digestion and nutrient absorption but also is considered as the largest immune organ. The intestinal mucosal immune system consists of mucosa associated lymphoid tissues (e.g., pai lymph nodes and mesenteric lymph nodes), effector lymphocytes dispersed between the lamina propria and intestinal epithelium, and corresponding effector molecules. The latter effectively monitor antigens passing through the intestinal tract, such as bacteria, viruses, and large proteins in food, to activate a mucosal immune response. Immunoglobulin IgA^+^, IgG^+^, and IgM^+^ cells play an important role in maintaining intestinal mucosal immune system homeostasis by secreting immunoglobulins (1, 2).

Immunoglobulins are important effector molecules in the humoral immune response to pathogens. They kill pathogens and limit their proliferation through, for example, conditioning, antibody-dependent cell-mediated cytotoxicity, and complement activation (3). They are also involved in regulating and coordinating immune responses by binding to antigens and to Fc receptors on immune cells. Further, they initiate and regulate the inflammatory and cytotoxic effects of various immune cells (4). Immunoglobulins are classified as IgA, IgG, IgM, IgD, and IgE. IgG is the main class of immunoglobulins produced during the immune response to foreign antigens and effectively protects the body (5, 6). IgG has a longer half-life and is more abundant and more effective in protecting the immune system than the other classes (7). IgG plays an important role in disease prevention in animals (8, 9). IgG binds to antigens and to a wide range of cells, such as phagocytes, lymphocytes, and mast cells (10). In mammals, IgG is the only antibody capable of passing through the placenta, completing trans-mammalian intestinal epithelial and placental transport via the neonatal Fc receptor for effective neonatal protection (11). Bovine IgG prevents inflammatory reactions induced by gastrointestinal infections, upper respiratory infections, and lipopolysaccharides (12). In calves, IgG is not only very important for the prevention of diarrhoea (13), but also has been associated with hypertension (14), hypogammaglobulinaemia (15), and nephritis. In addition, IgG deposition is associated with the balance of helper T-cells (Th)1/2 cells (16). IgG levels are accurate markers of the extent of bacterial and viral infections, and high levels of IgG provide long-lasting immunity to the host. Therefore, studying IgG is an effective means for determining host immune defences.

Alternative feed additives can provide a nutritional strategy to effectively prevent metabolic disorders in ruminants by improving the metabolic and immune status of animals (17). Oregano essential oil (OEO) is a plant oil extracted from oregano. Its major components are carvacrol and thymol (18). In animals, OEO can promote growth, exerts antioxidant, antibacterial, and anti-inflammatory properties, and is a natural alternative to antibiotics. OEO can promote gastrointestinal tract development (19), and effectively reduces the incidence of mastitis and diarrhoea in Holstein cows and improves their health (20). Further, OEO can improve the growth and slaughter performance of Holstein bulls, improve their meat quality, and enhance their antioxidant capacity (21). Wang et al. (22, 23) showed that adding OEO to the feed increased IgG levels and promoted postpartum recovery in dairy cows. Jia et al. (24, 25) found that OEO supplementation reduced pro-inflammatory interleukin 6 (IL-6) serum levels in Mongolian goats and sheep, thus alleviating inflammatory responses. However, the effects of OEO on intestinal mucosal immunity in Holstein dairy bulls remain unclear. Calves, especially male calves, play an important role in the herd and ensuring the health and production performance of calves is vital for dairy farms (26). In intensive feeding, the immune system tends to be weakened, so the use of antibiotics to strengthen the immunity of cattle is very necessary (27). Therefore, we assessed the effects of OEO dietary supplementation on IgG expression and the number of IgG^+^ plasma cells residing in the jejunum, ileum, and colon of Holstein bulls. Our aim was to provide evidence for further research on the mechanism of OEO, with the ultimate goal to improve the production performance of Holstein bulls.

## 2 Materials and methods

### 2.1 Test materials

Twelve healthy Holstein dairy bulls of approximately 10 months of age and with similar body weights (345.19 ± 3.89 kg) were obtained from Huarui Agricultural Company, Minle County, Zhangye City, Gansu Province, China. The animals were randomly equally divided into two groups. The control (CK) group was fed a basal ration [the nutrient composition of the basal diet is presented in Supplementary Table S1 (NRC, 2007)], and in the OEO group, OEO (Ralco Inc., Marshall, MN, United States) was added to the basal ration [20 g/head/day, based on previous research findings by our team (28)]. At the end of the 300-day feeding trial, the bulls were slaughtered after 24 h of fasting and 2 h of water fasting. Tissue samples were collected from the jejunum, ileum, and colon. Part of the samples was fixed by immersion in 4% formaldehyde solution, and the other part was stored at −80°C until analysis.

### 2.2 Haematoxylin-eosin staining

Paraffin sections (4 μm) were routinely prepared and deparaffinised to water. The sections were stained with haematoxylin for 6 min, rinsed with tap water for 30 min, subjected to hydrochloric acid alcohol differentiation for 10 s, and washed with tap water for 30 min for the colour to return to blue. Then, the sections were stained with eosin for 8 min and subjected to gradient alcohol dehydration and neutral gum sealing. Finally, the sections were scanned using a pathology slide scanner (Model No. DX1, Sruidi Medical Technology Co., Ltd., Shandong, China) and photographed to observe pathological changes in the jejunum, ileum, and colon.

### 2.3 Immunohistochemistry

The SABC immunohistochemical staining kit (Product No. SA1020) was used. Paraffin sections (4 μm) were routinely prepared and deparaffinised to water. They were subjected to antigen repair (citrate heat repair) for 10–15 min, cooled down to room temperature, and washed with distilled water. The sections were incubated in 3% H_2_O_2_ deionised water at room temperature to eliminate endogenous peroxidase activity and then washed three times with distilled water for 5 min each time. Drops of 5% normal goat serum blocking solution were added, and the sections were incubated at room temperature for 30 min to reduce non-specific binding. Primary antibodies IgG (Item No. ab6927, Abcam, United States) were added at a dilution of 1:600 (determined as the optimal working concentration among multiple dilutions tested), and the sections were incubated at 37°C for 1–2 h or at 4°C overnight (which had the best effect). As a negative blank control, we used phosphate-buffered saline (PBS) instead of the primary antibodies. The sections were washed three times with PBS for 5 min each time. Then, we added biotin-labelled secondary antibody (goat anti-mouse/rabbit IgG) working solution (2 mg/1 mL), and the slides were incubated at 37°C for 30–40 min and then rinsed with PBS three times for 5 min each time. Horseradish peroxidase-labelled streptavidin working solution (2 mg/1 mL) was added dropwise, and freshly prepared diaminobenzidine chromogen solution was added for colour development. The degree of colour development was monitored under a microscope. Colour development was terminated by washing with water. The sections were washed with distilled water wash, stained with haematoxylin for 1 min, and washed with water again. After gradient alcohol dehydration, the sections were sealed with neutral gum. The immunohistochemically stained sections were scanned and photographed using a pathology slide scanner. For each section, IgG^+^ cells in 10 random fields of view were counted, and densities were calculated using Image-ProPlus 6.0.

### 2.4 Enzyme-linked immunosorbent assay

Proteins were extracted using a whole protein extraction kit (BL521A, Solarbio technology Co., Ltd., Beijing, China). One gram of tissue sample (for samples, the fresh weight is required to be maintained, and the weight in the sample is not less than 50 mg, generally based on 1 g) was weighed and added to a lysis buffer (containing phosphatase inhibitor, protease inhibitor, and PMSF) on ice. The solution was mixed well and grounded with 2 mm magnetic beads until there were no obvious tissue pieces. The lysates were centrifuged at 4°C 12,000 × g for 30 min. The supernatants were aspirated into new centrifuge tubes and mixed again with 2 mm magnetic beads to ensure adequate grinding, and then centrifuged at 12,000 × g for 30 min at 4°C. The supernatants were aspirated into new centrifuge tubes. Protein contents were determined using a BCA protein concentration kit (BL521A). Finally, IgG^+^ protein concentrations in the jejunum, ileum, and colon were determined in using a Bovine IgG Enzyme Immunoassay Kit (YJ330698; Shanghai Enzyme-linked Biotechnology Co., Ltd., Shanghai, China) in strict accordance with the manufacturer’s instructions.

### 2.5 Statistical analysis

Statistical analyses were performed using IBM SPSS 26.0 software. The results are expressed as mean ± standard error. Means of two groups were compared using independent *t*-tests, and means of multiple groups were compared using one-way ANOVA. Differences were considered statistically significant at *p* < 0.05.

## 3 Results

### 3.1 Histological findings

**Figure 1:**
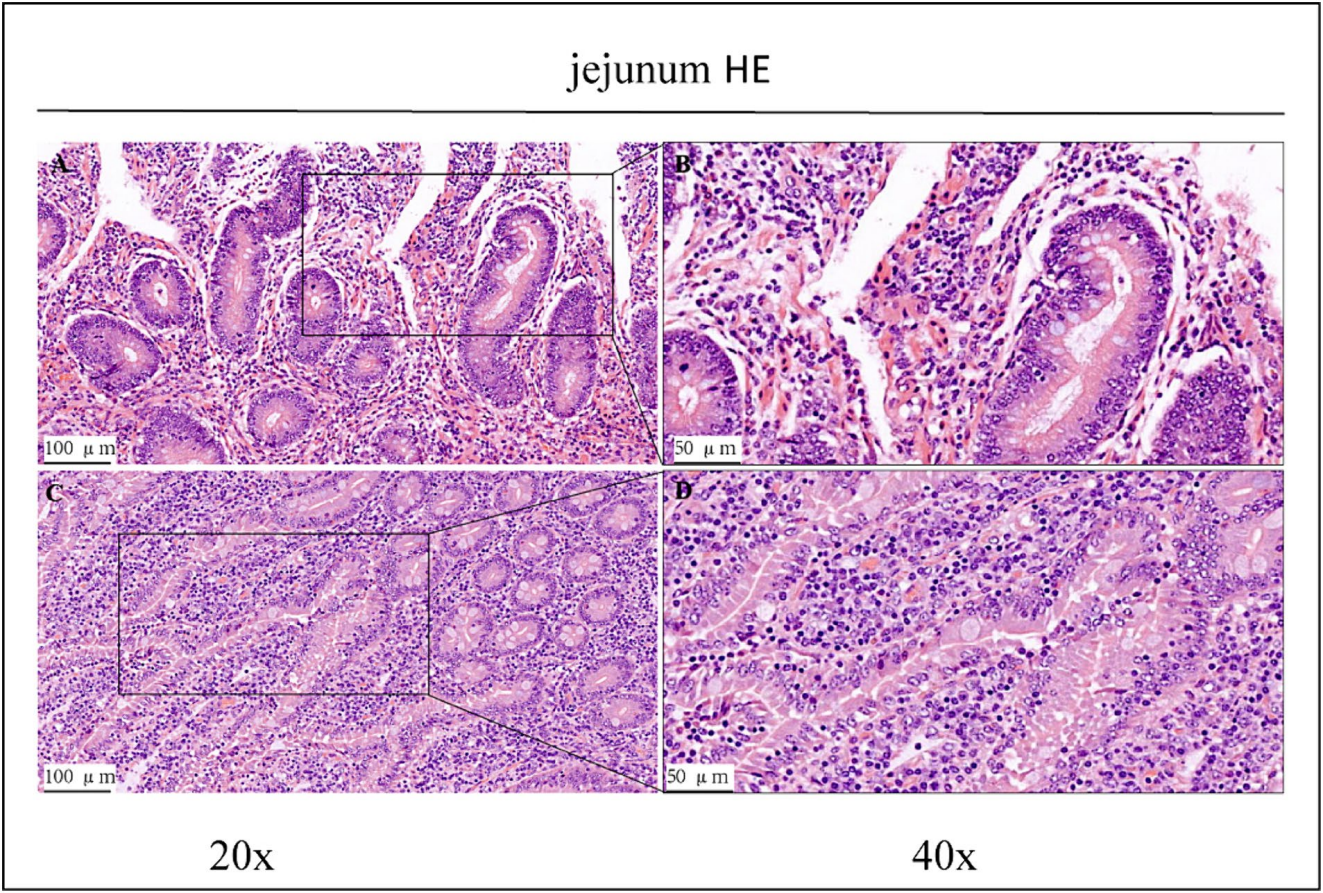
HE staining results of jejunum in CK group and OEO group (EO). **(A,B)** Are the CK group. **(C,D)** Are OEO group (EO). And the image on the right (scale, 50 μm) is a local magnification from the image on the left (scale, 100 μm).

**Figure 2:**
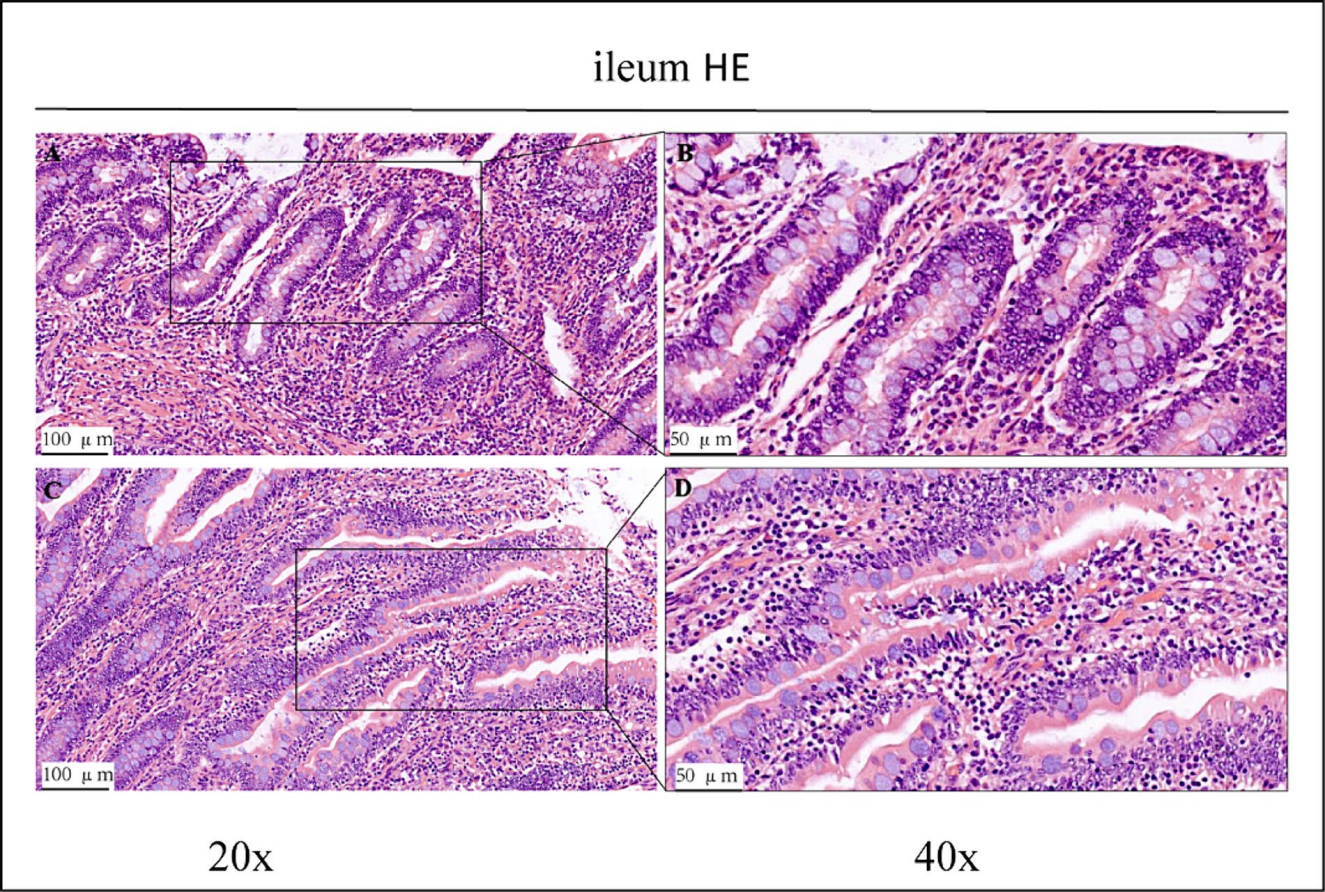
HE staining results of the ileum of control group (CK) and OEO group (EO). **(A,B)** Are the control group. **(C,D)** Are OEO group. And the image on the right (scale, 50 μm) is a local magnification from the image on the left (scale, 100 μm).

**Figure 3:**
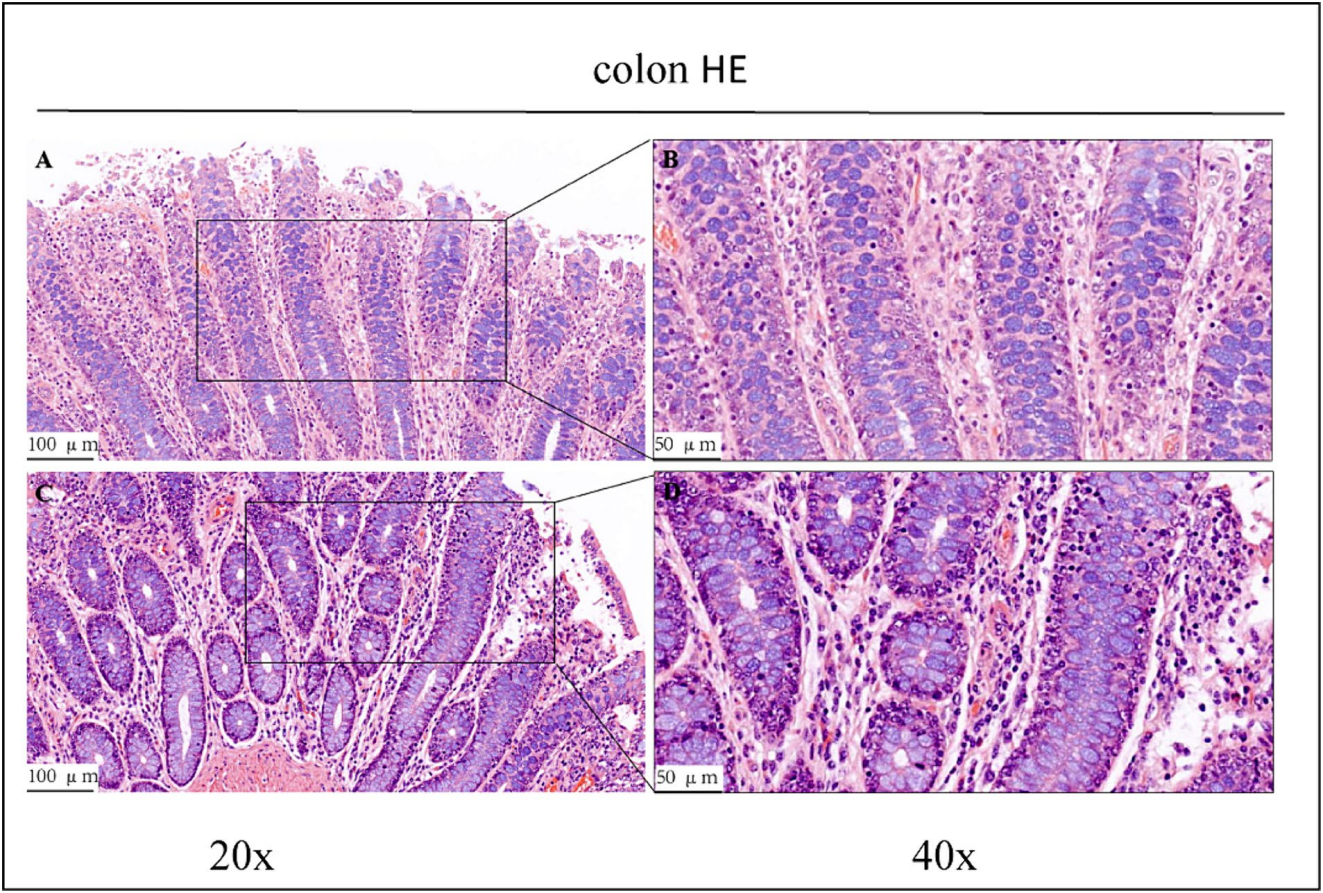
HE staining results of the colons of control group (CK) and OEO group (EO). **(A,B)** Are the control group. **(C,D)** Are oregano essential oil group. And the image on the right (scale, 50 μm) is a local magnification from the image on the left (scale, 100 μm).

Haematoxylin/eosin staining revealed inflammatory cell infiltration with varying degrees of intestinal villi detachment in some parts of the intestinal tract, as shown in Figures 1–3. The findings suggested that the experimental animals may have suffered from inflammation. Differences in inflammatory characteristics and the degree of inflammation between the CK and OEO groups could not be accurately determined, and further experimental verification is required.

### 3.2 Immunohistochemical findings

**Figure 4:**
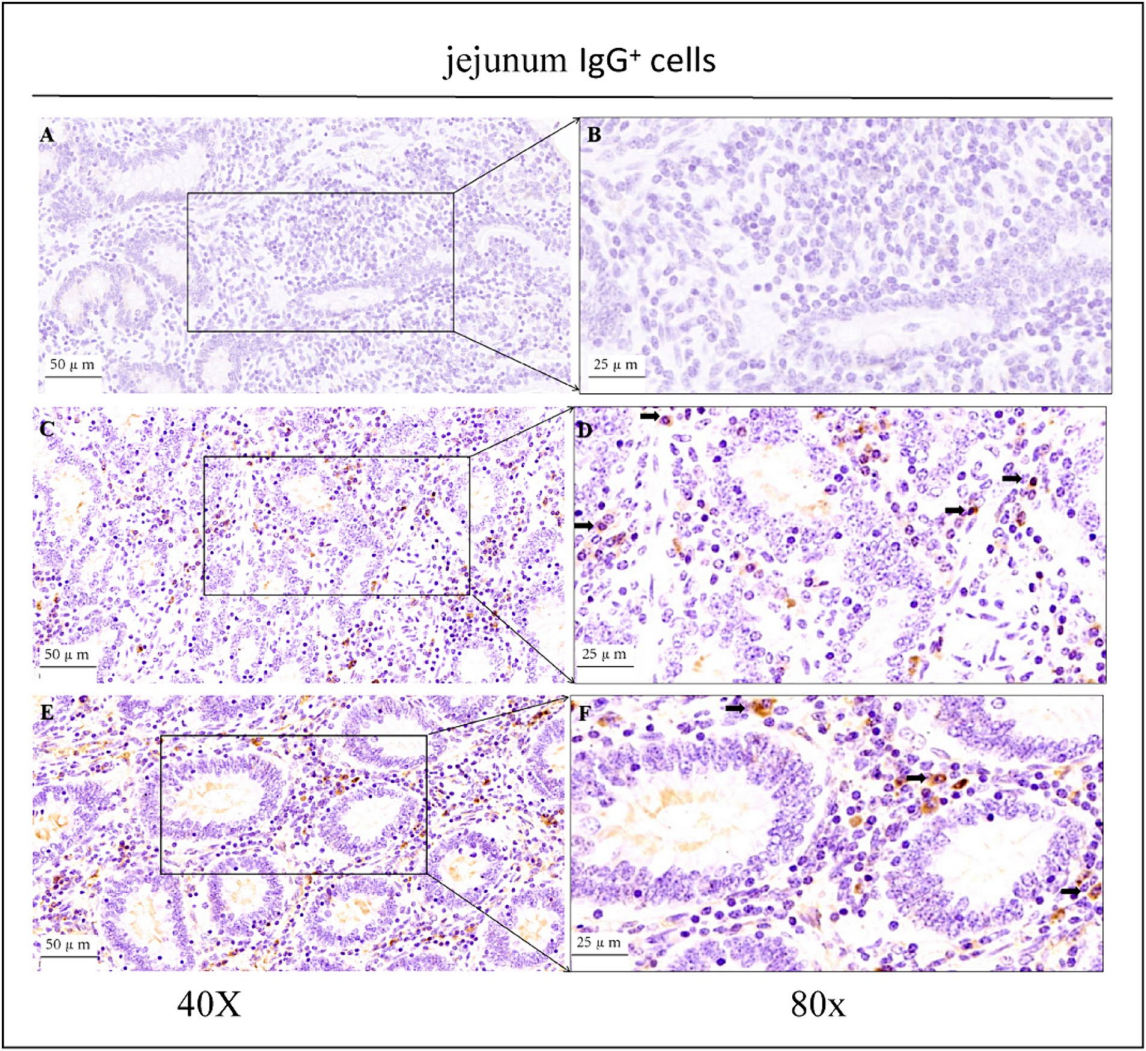
Distribution of IgG^+^ cells in the jejunum of depopulated Holstein bulls. **(A,B)** Are negative controls. **(C,D)** Are controls. **(E,F)** Are OEO groups. Arrows indicate IgG^+^ cells, and the right image (scale bar, 25 μm) is a magnification of the left image (scale bar, 50 μm).

**Figure 5:**
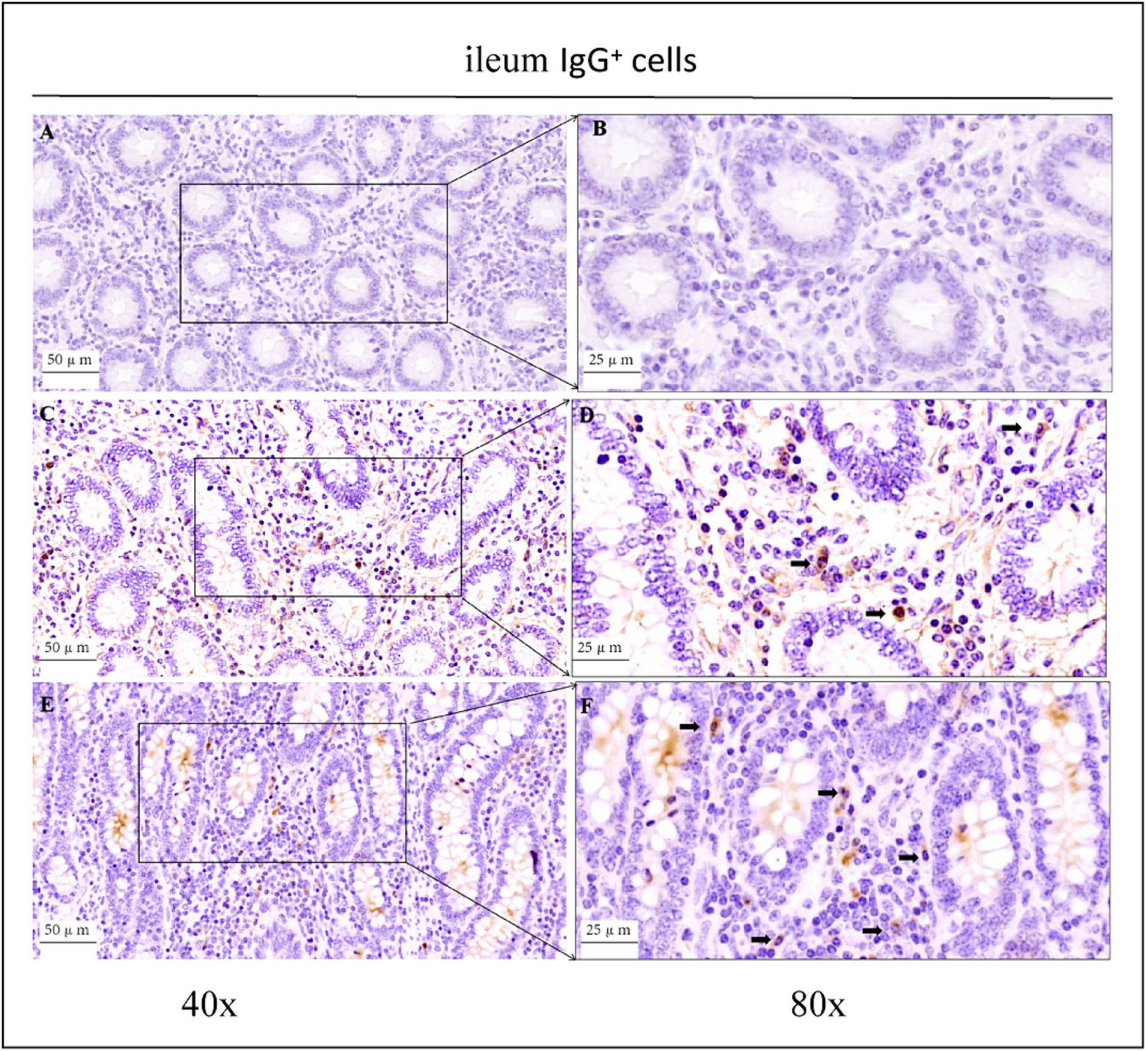
Distribution of IgG^+^ cells in the ileum of depopulated Holstein bulls. **(A,B)** Are negative controls. **(C,D)** Are controls. **(E,F)** Are OEO groups. Arrows indicate IgG^+^ cells, and the right image (scale bar, 25 μm) is a magnification of the left image (scale bar, 50 μm).

**Figure 6:**
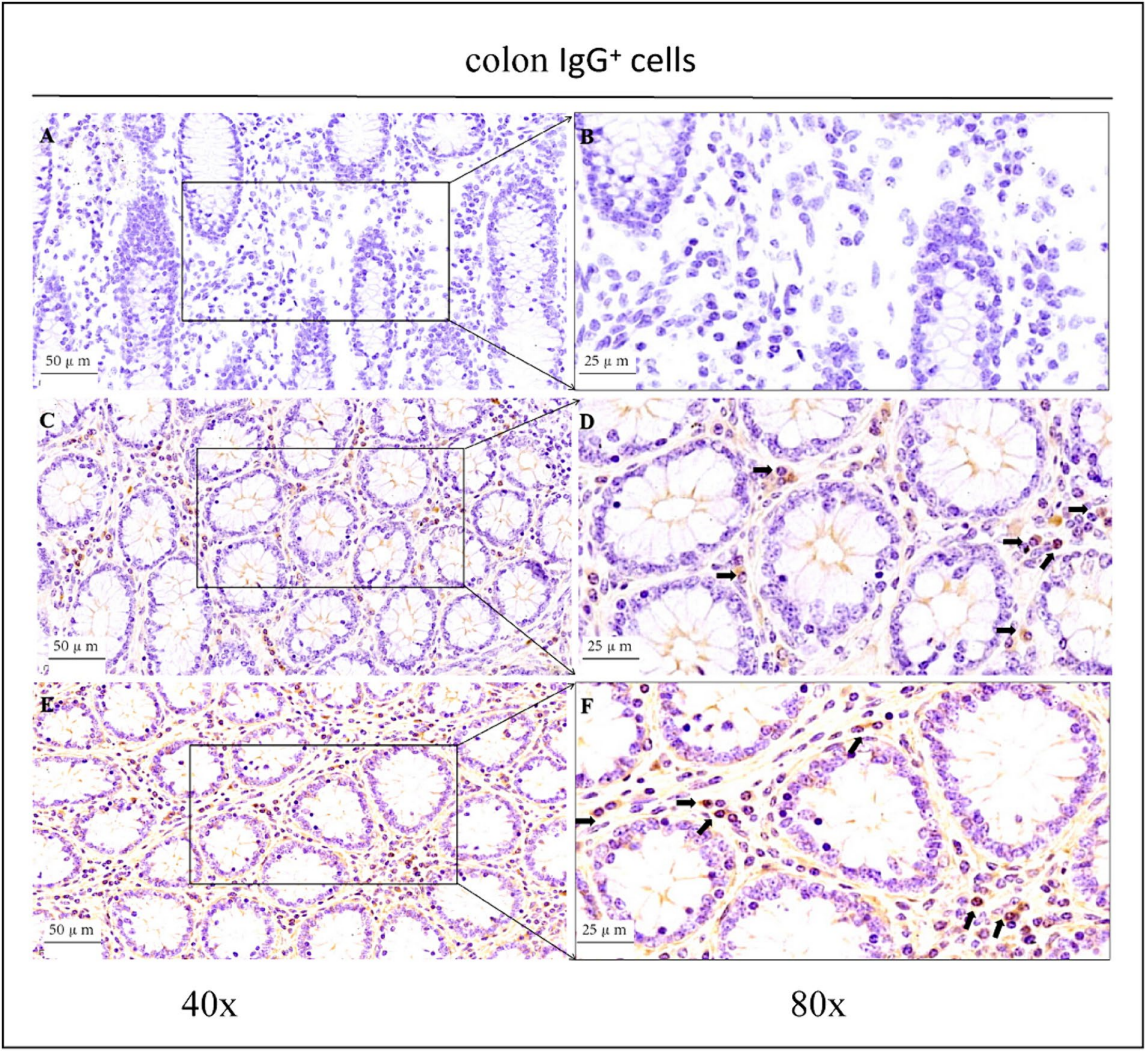
Distribution of IgG^+^ cells in the colon of depopulated Holstein bulls. **(A,B)** Are negative controls. **(C,D)** Are controls. **(E,F)** Are OEO groups. Arrows indicate IgG^+^ cells, and the right image (scale bar, 25 μm) is a magnification of the left image (scale bar, 50 μm).

As shown in the microscopic images in Figure 2, secretory cells were clearly visible, and their shapes were largely oval. Their cytoplasm stained brownish-yellow, and the nuclei were located on one side, and wheel-shaped. IgG^+^ cells were distributed in the jejunum, ileum, and colon, and their distribution was different in different intestinal segments. In the jejunum, they were mainly distributed in the lamina propria of the intestinal villi and of the basal part of the villi (Figure 4). In the ileum, IgG-secreting cells were mainly distributed in the lamina propria of the villi (Figure 5). In the colon, IgG-secreting cells were predominantly found in the lamina propria around the intestinal glands and a small amount was observed in the lamina propria of the intestinal villi (Figure 6). The cells were quite numerous and distributed diffusely throughout the intestine.

**Figure 7:**
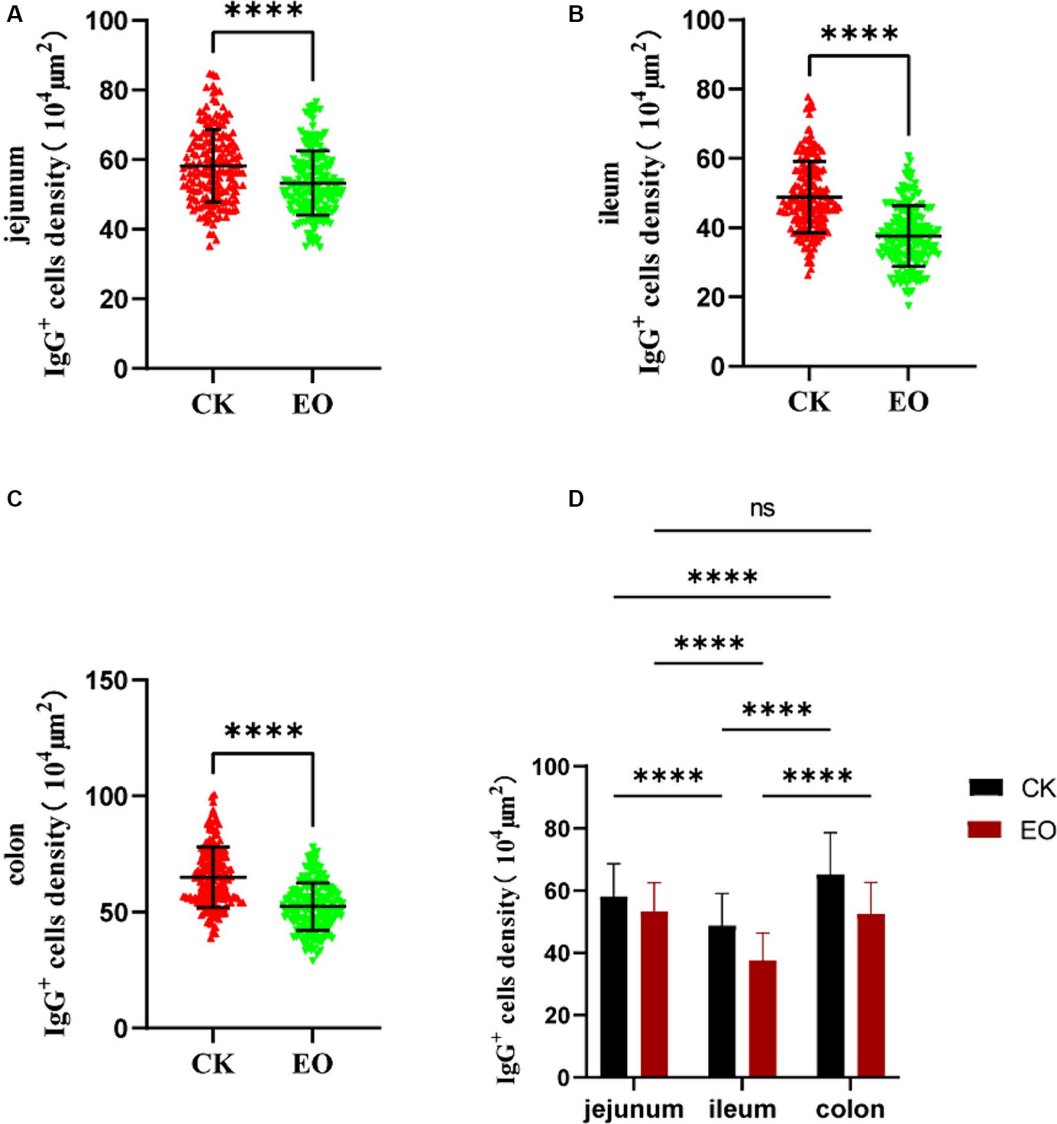
Effect of addition of oregano essential oil on IgG^+^ cell density in jejunum, ileum, and colon. **(A)** The effect of oregano essential oil on Holstein bull jejunum IgG^+^ cell density. **(B)** Effect of OEO on IgG^+^ cell density in the ileum of Holstein bulls. **(C)** Effects of oregano essential oil on colon IgG^+^ cell density in Holstein bulls. **(D)** Comparison of positive cells in three intestinal segments between OEO group (EO) and control group (CK). [OEO group (EO); control group (CK) “**” indicates statistically significant difference (*p* < 0.01)]. “****” indicates a significant difference.

The patterns of change in IgG-secreting cells among the three intestinal segments were largely the same (*p* > 0.05) in the CK and OEO groups. The density of IgG^+^ cells in all three intestinal segments was significantly reduced in the OEO group compared to the CK group (Figures 7A–C). As shown in the figure, there was no significant change in the cellular distribution of IgG^+^ cells in the OEO group compared to the CK group. In the jejunum, the IgG^+^ cell density was reduced by 8.52% in the OEO group compared to the CK group, in the ileum by 22.87%, and in the colon by 19.45% (Table 1). As shown in Figure 2, IgG secretory cells were significantly less distributed in the ileum than in the jejunum and colon in both the CK and EO groups, whereas the numbers of these secretory cells in the jejunum and colon were similar (*p* > 0.05) (Figure 7D). Thus, dietary supplementation of OEO may inhibit the inflammatory response in the intestine and lower the level of IgG^+^ cells to normal.

**Table 1:** Distribution of IgG in different intestinal segments of Holstein bulls.

Items	CK group	OEO group	*p*-value	Decrease rate (%)
Jejunum/10^4^μm^2^	58.24 ± 0.78	53.28 ± 0.69	<0.0001	8.52
Ileum/10^4^μm^2^	48.79 ± 0.77	37.63 ± 0.66	<0.0001	22.87
Colon/10^4^μm^2^	65.03 ± 0.98	52.38 ± 0.76	<0.0001	19.45

Differences in IgG-secreting cells among different sites in the intestine within the same group were analysed by one-way ANOVA [different letters indicate significant differences (*p* < 0.05)], and differences in IgG-secreting cells at the same site between the CK and OEO groups were analysed using independent samples *t*-tests (**p < 0.01). Reduction rate = (*X* − *Y*)/*X* * 100% (*X* is the density of positive cells in the CK group; *Y* is the density of positive cells in the OEO group).

### 3.3 Effect of OEO on IgG^+^ expression levels in the jejunum, ileum, and colon

**Figure 8:**
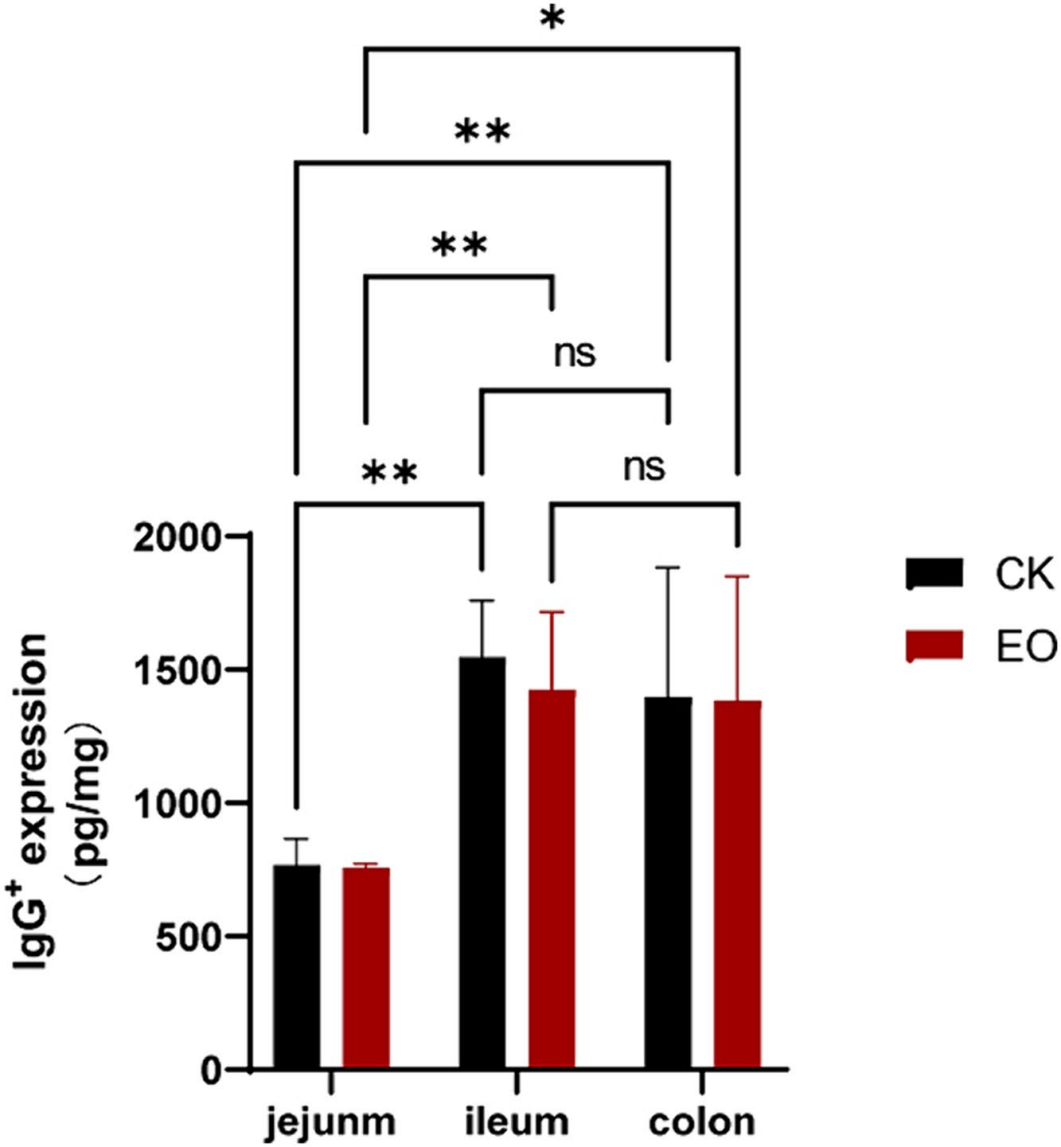
Effect of the OEO supplementation on IgG expression in the intestines of Holstein bulls OEO group (EO), control group (CK), [(**p < 0.01), (*0.01 < *p* ≤ 0.05)].

In enzyme-linked immunosorbent assays (ELISAs), serial dilutions of known standards are used to generate a standard curve, generally optical density versus concentration, from which the exact amount of the target in an unknown sample can be calculated. ELISA results showed that the expression level of IgG followed a similar trend as IgG^+^ cell density (Table 2), and that OEO supplementation reduced IgG expression in the intestinal tract of Holstein dairy bulls (Figure 8). The differences in IgG^+^ cells among the three intestinal segments did not correspond to the differences in expression, as the lowest expression was found in the jejunum.

**Table 2:** IgG expression levels in jejunum, ileum, and colon.

Items	CK group	OEO group	*p*-value
Jejunum/(pg/mg)	767.17 ± 40.57	758.20 ± 6.06	0.85
Ileum/(pg/mg)	1546.82 ± 94.98	1425.80 ± 118.21	0.45
Colon/(pg/mg)	1396.83 ± 198.37	1384.78 ± 208.05	0.96

This result was obtained from an independent samples *t*-test, *p* < 0.05, indicating a significant difference.

## 4 Discussion

Histopathological analyses revealed varying degrees of damage to the jejunum, ileum, and colon of Holstein dairy bulls in the CK group (Figure 1). In intensive production, high-concentrate rations are often used to meet the nutritional needs of cattle for fattening and milk production and to achieve high productivity in the short term. In cows, high-concentrate diets are often associated with a large number of inflammatory cells infiltrating the mammary glands, suggesting that the feeding of high-concentrate diets induces inflammatory responses in the mammary glands of cows (29). Further, prolonged high-concentrate feeding can lead to acidosis and cause damage to the digestive tract epithelium of the host, thus affecting its digestive and immune capabilities and consequently, its performance (30–33). High-concentrate diets induce the activation and epigenetic modification of NF-κB and MAPK signalling pathways in mammary tissues of dairy cows, thus promoting pro-inflammatory cytokine gene expression, which ultimately leads to inflammatory responses in the mammary tissues (34). Therefore, we conclude that the Holstein dairy bulls in the CK group in this study had some intestinal damage due to long-term high-concentrate feeding.

IgG, as the most abundant immunoglobulin in serum, is the major anti-infection immune antibody. Therefore, it can be used as an important indicator for the diagnosis of inflammation. Immunohistochemical staining revealed the presence of a large number of IgG-secreting cells in the lamina propria of the intestinal mucosa of Holstein dairy bulls, suggesting that IgG plays an important role in protecting the intestinal mucosa. Both immunohistochemical staining and ELISA results showed that the addition of OEO significantly reduced IgG^+^ plasma cell numbers and IgG expression levels in all intestinal segments. This may be because when inflammation occurs in the organism, the binding of antigens and antibodies triggers massive immunoglobulin production, leading to abnormally high immunoglobulin levels in the organism (35, 36). OEO is rich in phenolic substances that have anti-inflammatory properties and reduce IgG secretion to normal levels by reducing IgG secretion to achieve an anti-inflammatory effect. These anti-inflammatory properties of OEO enable it to reduce the inflammatory response caused by damage to the intestinal mucosal barrier and to promote intestinal barrier function and mucosal barrier integrity. Essential oils have a protective effect on the intestinal mucus layer, reducing the exposure and adsorption of harmful substances. Plant essential oils can improve intestinal barrier function and reduce the degree of inflammation by regulating the expression levels of genes related to intestinal inflammation and other related gene (37). OEO exerts anti-inflammatory effects by regulating the expression of inflammatory factors such as IL-1β, IL-6, and tumour necrosis factor-α (38–40), which in turn reduce the risk of intestinal inflammation in Holstein dairy bulls. The level of intestinal IgG-secreting cells varies somewhat between species, providing a basis for different parts of the digestive tract to function to different degrees. The presence of numerous IgG-secreting cells in a particular site indicates a high capacity to prevent the invasion of toxic and harmful substances into the intestinal mucosal epithelium in that site (41).

In conclusion, dietary supplementation of OEO did not alter the spatial distribution of IgG^+^ plasma cells in the jejunum, ileum, and colon of Holstein dairy bulls, but it did significantly reduce the number of IgG^+^ plasma cells and IgG content in the three segments of the intestine. Our findings indicate that OEO can alleviate intestinal inflammation in Holstein dairy bulls and exerts anti-inflammatory action by reducing immunoglobulin levels in the intestinal tissues to enhance body immunity. Therefore, OEO can be used as a feed additive in cattle breeding to promote host immunity, thus effectively enhancing production performance. The detailed mechanism underlying the regulation of IgG^+^ plasma cell production by OEO requires further study.

## Data availability statement

The original contributions presented in the study are included in the article/Supplementary material, further inquiries can be directed to the corresponding author.

## Ethics statement

The animal study was approved by Animal Policy and Welfare Committee of Gansu Agricultural University (Ethic approval file No. GSAU-Eth-AST-2022-035). The study was conducted in accordance with the local legislation and institutional requirements.

## Author contributions

MX: Conceptualization, Data curation, Formal analysis, Investigation, Methodology, Project administration, Software, Validation, Writing – original draft, Writing – review & editing. WZ: Formal analysis, Methodology, Supervision, Validation, Writing – review & editing. FK: Conceptualization, Investigation, Supervision, Writing – review & editing. BW: Data curation, Resources, Writing – review & editing. JP: Data curation, Writing – review & editing. JS: Methodology, Software, Writing – review & editing. QL: Data curation, Methodology, Software, Writing – review & editing. PH: Methodology, Software, Writing – review & editing. YM: Methodology, Writing – review & editing. QC: Methodology, Writing – review & editing. ZZ: Methodology, Writing – review & editing. ZL: Conceptualization, Data curation, Formal analysis, Methodology, Project administration, Software, Writing – review & editing.
