# Supplementary material for: Effect of oregano essential oil on intestinal immunoglobulin G in Holstein dairy bulls
Source: Front Vet Sci. 2024 Jul 29;11:1382396. doi: 10.3389/fvets.2024.1382396 (PMC11319842; doi:10.3389/fvets.2024.1382396)
Supplement: Supplementary file 1 [file Table_1.DOCX]

Supplementary Material

**Table S1.** Composition and nutrient levels of the basal diet, % of DM

| Ingredients | content |
| --- | --- |
| Corn straw silage | 17.18 |
| Corn straw | 8.86 |
| Corn | 44.31 |
| Bran | 3.69 |
| Concentrates | 8.86 |
| Soybean meal | 2.58 |
| Cottonseed meal | 3.69 |
| Rapeseed meal | 1.85 |
| Corncob | 4.43 |
| Malt sprout | 1.85 |
| Oil | 0.74 |
| Premix ^1)^ | 1.85 |
| NaCl | 0.07 |
| NaHCO_3_ | 0.04 |
| Total | 100 |
| Nutrient levels ^2)^ |  |
| DM | 88.30 |
| CP | 13.49 |
| DE/(MJ/kg) | 12.85 |
| NDF | 24.97 |
| Ca | 0.51 |
| P | 0.31 |

^1)^ The premix provided the following per kg of the diet: 100,000 IU of vitamin A, 800,00 IU of vitamin D, 550 IU of vitamin E, 1800 mg of Mn, 2100 mg of Zn, 1200 mg of Fe, 300 mg of Cu, 20 mg of I, 8 mg Se.

^2)^ Nutrient levels were measured value.
